# A qualitative co-design-based approach to identify sources of workplace-related distress and develop well-being strategies for cardiovascular nurses, allied health professionals, and physicians

**DOI:** 10.1186/s12913-024-10669-x

**Published:** 2024-02-26

**Authors:** Ahlexxi Jelen, Rebecca Goldfarb, Jennifer Rosart, Leanna Graham, Barry B. Rubin

**Affiliations:** 1 Peter Munk Cardiac Centre, Toronto General Hospital, University Health Network, 585 University Avenue LPMB 119 K, M5G 2N2 Toronto, ON Canada; 2 Goldfarb Intelligence Marketing, Toronto, Canada; 3 Healthcare Human Factors, Toronto General Hospital, University Health Network, Toronto, Canada; 4 Office of Professional Practice & Policy, Toronto General Hospital, University Health Network, Toronto, Canada; 5 Division of Vascular Surgery, Peter Munk Cardiac Centre, University Health Network, Toronto, Canada

**Keywords:** Occupational stress, Distress, Burnout, Well-being, Workplace factors, Intervention strategies, Nurses, Allied health professionals, Physicians

## Abstract

**Objective:**

Clinician distress is a multidimensional condition that includes burnout, decreased meaning in work, severe fatigue, poor work–life integration, reduced quality of life, and suicidal ideation. It has negative impacts on patients, providers, and healthcare systems. In this three-phase qualitative investigation, we identified workplace-related factors that drive clinician distress and co-designed actionable interventions with inter-professional cardiovascular clinicians to decrease their distress and improve well-being within a Canadian quaternary hospital network.

**Methods:**

Between October 2021 and May 2022, we invited nurses, allied health professionals, and physicians to participate in a three-phase qualitative investigation. Phases 1 and 2 included individual interviews and focus groups to identify workplace-related factors contributing to distress. Phase 3 involved co-design workshops that engaged inter-professional clinicians to develop interventions addressing drivers of distress identified. Qualitative information was analyzed using descriptive thematic analysis.

**Results:**

Fifty-one clinicians (24 nurses, 10 allied health professionals, and 17 physicians) participated. Insights from Phases 1 and 2 identified five key thematic drivers of distress: inadequate support within inter-professional teams, decreased joy in work, unsustainable workloads, limited opportunities for learning and professional growth, and a lack of transparent leadership communication. Phase 3 co-design workshops yielded four actionable interventions to mitigate clinician distress in the workplace: re-designing daily safety huddles, formalizing a nursing coaching and mentorship program, creating a value-added program e-newsletter, and implementing an employee experience platform.

**Conclusion:**

This study increases our understanding on workplace-related factors that contribute to clinician distress, as shared by inter-professional clinicians specializing in cardiovascular care. Healthcare organizations can develop effective interventions to mitigate clinician distress by actively engaging healthcare workers in identifying workplace drivers of distress and collaboratively designing tailored, practical interventions that directly address these challenges.

**Supplementary Information:**

The online version contains supplementary material available at 10.1186/s12913-024-10669-x.

## Introduction

Workplace distress is widespread problem faced by healthcare workers with significant consequences for patients, providers, and healthcare systems [1]. Distress is a composite of multiple clinically relevant dimensions that include burnout, decreased meaning in work, severe fatigue, poor work–life integration, low quality of life, and suicidal ideation [2–4]. Prolonged exposure to work-related stressors increases the risk of burnout. Professional burnout is characterized by emotional exhaustion, depersonalization, and a sense of reduced personal accomplishment [5, 6]. For clinicians, burnout is intertwined with poor physical and mental health, and adversely effects the quality of care that they can provide. This results in increased medical errors, serious safety events, reduced patient satisfaction, and worse patient outcomes [7–10]. Moreover, clinician burnout has substantial economic impacts on healthcare systems due to high staff turnover, increased absenteeism, and decreased productivity [11, 12].

The underlying drivers of clinician distress in the workplace are multifaceted. Excessive workloads, increased job demands, chaotic work environments, limited control or flexibility, insufficient reward for effort, breakdown of community, and difficult patient encounters are among the primary sources of workplace stress [6, 13–16], all of which were exacerbated during the COVID-19 pandemic [17]. However, this problem exists regardless of the pandemic. Our previous research has shown that clinicians specializing in cardiovascular care experience varying levels of burnout and distress. Among them, the prevalence of burnout was 79% in nurses, 73% in allied health professionals, and 65% in physicians. Additionally, the prevalence of high distress was 78%, 56%, and 65% of nurses, allied health professionals, and physicians, respectively [18–20]. Among these clinician groups, the perception of unfair workplace treatment and inadequate staffing levels emerged as principal drivers of high distress [18–20]. Since drivers of distress vary across healthcare environments, interventions to ameliorate distress must be tailored to individual workplace settings. This is important as cultivating positive work environments can mitigate clinician distress while improving job satisfaction and the delivery of quality care [14].

As awareness for the prevalence and drivers of clinician burnout and distress increases, it is promising that healthcare organizations are starting to take action [1]. However, organization-wide assessment of clinician well-being and implementation of intervention strategies to mitigate burnout and distress are still lacking. This gap has been emphasized in a recent study [21], highlighting the critical need for healthcare organizations to adopt a comprehensive approach in both the assessment and promotion of clinician well-being. In our pursuit to develop effective interventions within our healthcare setting, our team built upon our previous research assessing the well-being of cardiovascular clinicians [18–20] to qualitatively explore the associations between well-being and workplace-related distress. This involved qualitatively studying the drivers of workplace-related distress through individual interviews and focus groups with cardiovascular nurses, allied health professionals, and physicians. Additionally, we used a co-design process with these clinician groups to collaboratively develop tailored interventions aimed to reduce distress and improve well-being in their workplace. Co-design is a collaborative process that actively involves stakeholders with lived experience, expertise, or knowledge throughout the design process to develop products, services, or solutions that more effectively meets their needs [22]. In a healthcare context, this process can be used to engage clinician stakeholders to improve their care environment [23, 24]. Combining the creative and empathetic aspects of design thinking with the structured approach of systems thinking when co-designing well-being interventions has also been suggested as a potential means to achieve more effective outcomes [24].

### Objectives

The objective of this qualitative investigation was two-fold:

1. Identify the factors that drive the perception of unfair treatment at work and elucidate other workplace-related factors that contribute to distress among nurses, allied health professionals, and physicians specializing in cardiovascular care.
2. Engage in a collaborative co-design process to develop potential interventions that would address sources of workplace distress identified by participating clinicians.

## Methods

### Setting, participants, and study design

We invited nurses, allied health professionals, and physicians specializing in cardiovascular care at a quaternary care network in Toronto, Canada to participate in a three-phase qualitative investigation on workplace distress. This investigation took place during the COVID-19 pandemic between October 2021 and May 2022. Clinicians were invited to participate in one or more qualitative phase of the research, including individual interviews (Phase 1), focus groups (Phase 2), or co-design workshops (Phase 3). We used a convenience sampling strategy to recruit clinicians that held a full-time permanent position in the Peter Munk Cardiac Centre (PMCC) at the University Health Network for a minimum of 18 months. Clinicians self-identified as potential participants or were nominated by colleagues or divisional leadership. The project team obtained verbal consent and clinicians were informed that participation was voluntary. Participants received an honorarium for each phase of the project that they participated in.

In each phase, qualitative discussions were conducted virtually via video conferencing and led by project team members with expertise in qualitative interviewing (RG, AC, JR). To minimize bias, ensure honest conversations, and diffuse power differentials, interviewers from the project team were non-PMCC employees. Interviews remained confidential with no individual results shared with the broader project team or members of the PMCC. Discussion guides for each phase were developed by our team for the purposes of this study and were used to facilitate discussions in each phase. These guides built upon insights from previous phases and were initially tested during individual interviews in Phase 1, which were jointly conducted by two leads (RG, AC, JR). Discussion guides for Phases 1–3 can be found in the supplement.

### Ethics

The University Health Network Research Ethics Board provided a waiver for the requirement for the research ethics approval for this project (QI ID#: 21–0271).

### Data collection

**Fig. 1.**
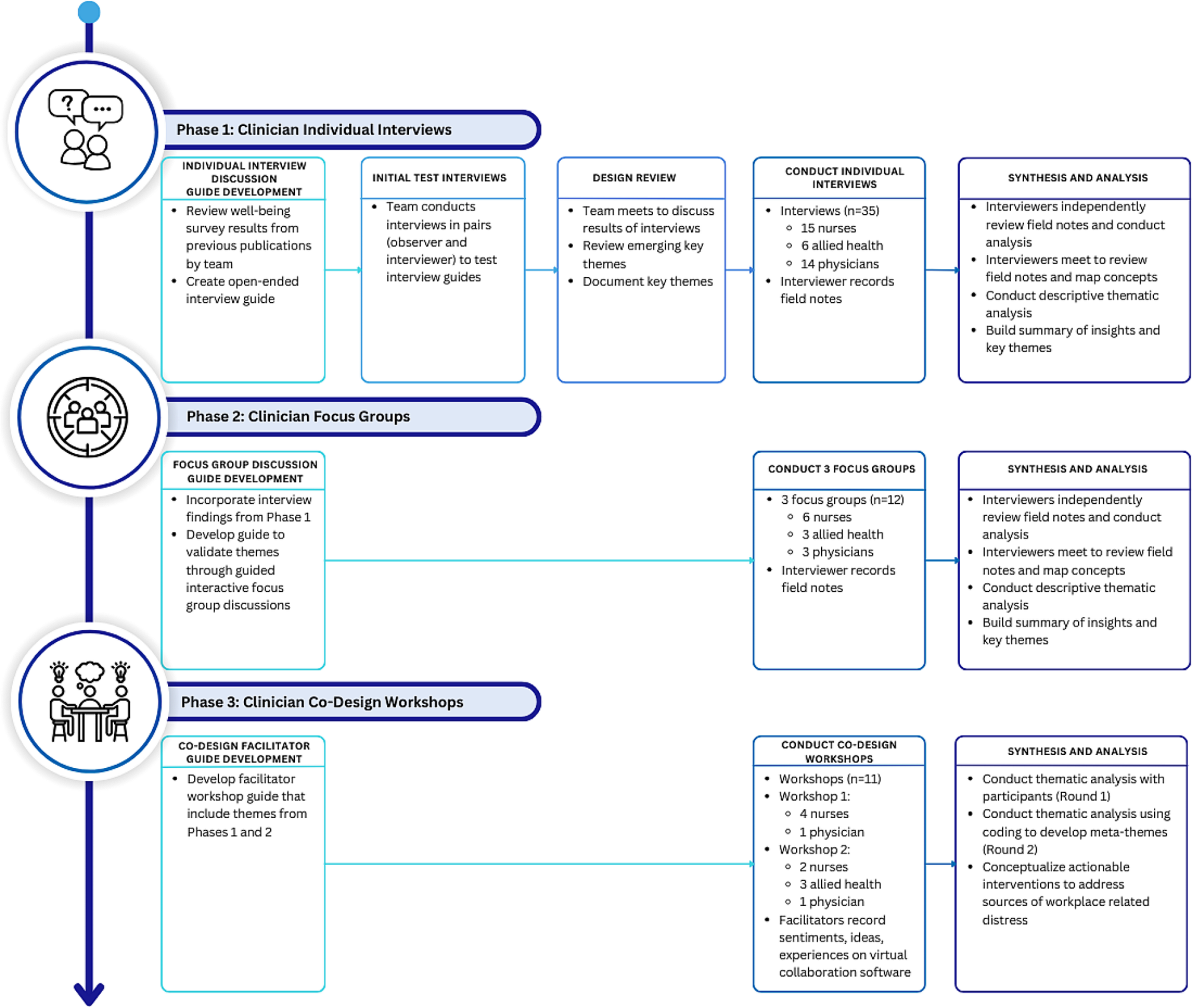
Steps of the three-phase qualitative investigation on workplace clinician distress

In Phases 1 and 2, we gathered qualitative insights from individual interviews and focus groups to understand the perceptions of nurses, allied health professionals, and physicians regarding unfair treatment and other workplace-related factors contributing to distress within their clinical environments. Interviewers used probing techniques to explore responses with participants in greater depth. Findings from each phase were used to build an adapted discussion guide for subsequent phases. Additionally, participants were provided with a summary of key insights and themes at the onset of each subsequent phase. Qualitative discussions and thematic findings were documented by interviewers (RG, AC, JR) using field notes and were reviewed at the start, mid-point, and end of Phases 1 and 2. Data saturation was determined when little or no relevant new information was found, or when information was repeated without adding any new understanding or contribution to a given theme [25]. In Phase 3, facilitators (JR, AS, DP) gathered sentiments, ideas, and experiences from participants through co-design workshops using virtual post-it notes on Miro, a virtual collaboration tool (https://miro.com/). Project team members (JR, AC, RG, AJ) then synthesized findings to conceptually design and prioritize actionable interventions. The three-phase qualitative approach is outlined in Fig. 1.

#### Phase 1: clinician individual interviews

To have honest conversations and diffuse power differentials that often occur in group interactions, individual clinician interviews were conducted. An open-ended discussion guide was used, and information was recorded using field notes. Interviews explored broad social contexts of work and personal life to understand perceptions and drivers of distress as it related to unfair treatment [18–20], along with a recent assessment of clinician well-being using the Well-Being Index [2–4]. Additional topics explored role and job function, racial bias and discrimination, workload, work–life balance, impact of the COVID-19 pandemic, and other challenges raised by participants during interviews.

#### Phase 2: clinician focus groups

In small focus groups of 3–6 participants, organized for nursing, allied health, and physician professions, participatory design activities and guided conversations were conducted. Interviewers facilitated groups using an adapted discussion guide and documented discussions using field notes. At the start of each focus group, participants were presented with a summary of key insights and themes. Participants were invited to share feedback on each identified theme and provided additional insights not covered in Phase 1. During each focus group, interviewers encouraged interactive discussions and thoughtful reflection to explore clinicians’ perceptions of unfair workplace treatment and other workplace-related factors contributing to distress, validating and building upon insights gathered during Phase 1.

#### Phase 3: clinician co-design workshops

We conducted two workshops with inter-professional clinicians using a co-design approach [22–24]. The goal of these workshops was to evolve findings from Phases 1 and 2 into actionable interventions aimed to reduce clinician distress and improve well-being. At the beginning of each workshop, the combined thematic findings from Phases 1 and 2 were shared with participants along with a series of stories to illustrate ways in which ideal interventions might come to life. The co-design approach used both design and systems thinking to encourage brainstorming of ideas and potential interventions [24]. As participants worked through the co-design activities, workshop facilitators captured their ideas, sentiments, or narrated experiences onto virtual post-it notes using a virtual collaboration tool. Each post-it note represented a data unit for analysis. Facilitators guided cross-disciplinary idea generation and refinement of potential interventions aimed at addressing the workplace drivers of distress identified during these workshops.

### Analysis

Qualitative information gathered in Phases 1 and 2 was analyzed using a qualitative descriptive design [26]. Qualitative descriptive analysis involved deductive and inductive processes [27] by gathering insights from pre-formed hypotheses and allowing new themes to emerge from the data [27, 28]. At the end of each phase, interviewers (RG, AC, JR) independently analyzed and coded qualitative information from field notes to identify themes and explore the relationships between them [27]. Subsequently, all interviewers (RG, AC, JR) met in a structured discussion environment to establish common themes by comparing and validating individual findings from their field notes. Using an iterative process, project team members (RG, AJ, JR, AC, AS) organized and synthesized findings using concept mapping and code interpretation [27, 29]. Findings were then shared with all members of the project team to build consensus on the existing and emerging themes identified. Key findings and themes were reviewed and validated with participants in each subsequent phase of this project. In Phase 3, information captured during the co-design workshops was analyzed through two rounds of synthesis. The first round was a thematic analysis conducted by workshop facilitators (JR, AS, DP) in partnership with participants. This allowed clinician stakeholders to ensure that the representation and interpretation of their ideas was accurate. A second round was conducted by the facilitators (JR, AS, DP), together after the workshop, using open and axial coding to create overarching meta-themes, each representing potential interventions that could address the identified drivers of workplace distress [27]. Each potential intervention was further refined by project team members (AJ, RG) to establish actionable interventions.

## Results

A total of 51 inter-professional clinicians participated in this study, including 24 nurses, 10 allied health professionals, and 17 physicians. Participant characteristics are reported in Table 1. In Phase 1, 35 individual semi-structured interviews were completed with 35 clinicians including 15 nurses, 6 allied health professionals, and 14 physicians. In Phase 2, three clinician-specific focus groups were conducted with 12 clinicians. Each focus group comprised of 6 nurses, 3 allied health professionals, and 3 physicians. In Phase 3, two inter-professional co-design workshops were conducted with 11 clinicians, 7 of whom previously participated in phases 1 or 2, and 4 of whom did not. Workshop 1 included 4 nurses and 1 physician. Workshop 2 included 2 nurses, 3 allied health professionals, and 1 physician.

**Table 1:** Participant Characteristics

Characteristics	N (%)
Clinical Discipline	
Nurses	24
Nurse Manager	1 (4)
Nurse Practitioner	3 (13)
Patient Care Coordinator	2 (8)
Registered Nurse	17 (71)
Allied Health Professionals	10
Occupational Therapist	1 (10)
Pharmacist	1 (10)
Physiotherapist	1 (10)
Respiratory Therapist	3 (30)
Sonographer	3 (30)
Speech Language Pathologist	1 (10)
Physicians	17
Anesthesiologist	3 (18)
Cardiologist	5 (29)
Cardiovascular Surgeon	3 (18)
Interventional Radiologist	1 (6)
Vascular Surgeon	2 (12)
Years of service	
18 months– 5 years	16 (31)
6–10 years	10 (20)
11–20 years	16 (31)
21–26 years	5 (10)
> 27 years	4 (8)

Findings from Phases 1 and 2 were organized into five major themes: (1) supportive inter-professional teams are desired to build an effective care community; (2) joy in work is paramount for clinician well-being and exceptional patient care; (3) unsustainable workloads are strongly linked to clinician distress; (4) professional growth and development are key to well-being and job satisfaction; and (5) open and transparent communication by leadership is a critical enabler of well-being. In Phase 3, clinician participants generated ideas for potential interventions from four themes synthesized from findings in Phases 1 and 2. The theme of unsustainable workload was excluded from workshop discussions, as it required additional financial and human resources to explore. Workshop input was synthesized into actionable interventions to address aspects of the work environment that contributed to clinician distress. Actionable interventions included redesigning daily unit safety huddles, establishing a nursing coaching and mentorship program, creating a value-added program e-newsletter, and implementing an employee experience platform.

### Key themes driving clinician distress that emerged from Phases 1 and 2

#### Supportive inter-professional teams are desired to build an effective care community

All clinicians expressed the desire to work as a cohesive and respectful care team. They stressed the need to strengthen positive inter-professional relationships to improve their work experiences and delivery of quality patient care. Unfair treatment and favouritism emerged as key challenges influencing both individual and team dynamics. Participants highlighted how disrespect and incivility within their teams led to unprofessional interactions, making it difficult to address issues in their care settings without sufficient resources or management support.

Nurses expressed their desire to be part of effective and respectful care teams that value their skills and insights. However, they encountered perceptions of unfair treatment, such as favouritism, that sometimes contradicted this desire. Some nurses felt invisible or excluded during informal meetings, while others felt powerless to bring about positive changes in their practice area. Nurses reported instances of mistreatment, including physicians not addressing them by name, receiving disrespectful e-mails, being yelled at by colleagues, or facing mockery by other nurses. They also noted unfair treatment of younger or less experienced peers. Nurses occasionally faced difficult or abusive patient encounters, leaving them feeling unsafe and uncertain about how to respond. They hesitated to seek support by colleagues or management. Many nurses expressed dissatisfaction with the lack of recognition for their hard work despite the expectation to give “110%.” They believed that equal and purposeful expressions of appreciation would significantly improve their sense of being valued and treated fairly.

Allied health professionals also faced issues related to respect and fairness. They described having mixed interactions with nurses and physicians, with some challenges integrating their specialized skills into the care team. They reported that some nurses and physicians lacked respect or understanding of their expertise. They felt it was essential for all team members to have a clear understanding of their roles for more effective delivery of team-based care.

Physicians acknowledged the importance of fostering positive inter-professional relationships. They identified broader reasons for teamwork challenges, including heavy workloads, insufficient training for inexperienced staff, and high turnover. Some female physicians pointed out gender biases and experienced differential treatment compared to their male counterparts. Physicians acknowledged that hospital environments can be stressful and that communication among team members was not always respectful. They also noted expressions of gratitude were often assumed rather than spoken. One physician said, *“It’s important to show appreciation. Talk. Introduce yourself. Remember to say thank you, good morning, and goodbye.”*

#### Joy in work is paramount for clinician well-being and exceptional patient care

All three clinician groups remarked on the importance of finding joy in work. They took pride in caring for people living with complex heart and vascular diseases at an institution rated among the highest in the world. While they felt satisfaction in making a positive impact on patients’ lives, consistently experiencing joy in work was a challenge. They often attributed the diminished sense of joy to a lack of time to reflect on meaningful patient care moments.

Nurses stressed the importance of making a positive impact on patients’ lives for finding joy in their work. They found joy in meaningful patient interactions, like offering words of encouragement or making them laugh. Nurses consistently reported that one of the most fulfilling parts of their profession was educating patients and enabling them to be active partners in their care planning. However, joy decreased when heavy workloads or staffing shortages limited bedside care, or when colleagues lacked respect or gratitude and showed favouritism. These situations were identified as sources of their distress.

Allied health professionals found joy in their work by positively impacting patients’ lives and educating them to manage their medical conditions. They also connected joy in work to opportunities for professional growth, such as mentorship, contributing to research, or taking on new roles that advanced their careers. Joy in work was said to diminish when they had insufficient time for patient care, limited opportunities for professional growth, or when their contributions on the team went unnoticed by their clinician colleagues. A perceived lack of respect, fairness, and gratitude was associated with reduced workplace joy and increased distress among this group.

Physicians found joy in work through achieving positive patient outcomes but said that experiencing joy was often not a personal reality. Like their colleagues, physicians experienced joy by helping patients lead better lives. *“Giving the gift of life.”* is special and unique to the role of healthcare providers one physician remarked. Others stated that *“The best part of my work is the patients. I enjoy hearing their stories.”* and *“It is so gratifying to see the look in a patient’s or loved one’s eyes. You can’t get this from closing a $100 million business deal.”* While they took prided in their professional accomplishments, physicians felt less personal fulfillment and experienced increased distress when there was limited hospital resources or support to deliver the best patient outcomes, especially during the pandemic.

#### Unsustainable workloads are strongly linked to clinician distress

Clinicians highlighted that shortages in frontline staffing had a significant impact on their ability to carry out their jobs effectively. Staffing shortages were identified as a primary reason for increased workloads, leading to daily fatigue and distress. Uncertainty about the future of the healthcare workforce was a primary concern among clinicians, and they were pessimistic about the organization or health system finding a resolution.

Nurses acknowledged that staffing shortages and turnover increased their workloads, which negatively affected their daily work experiences and ability to take time off. They reported feelings of unfair treatment and distress, especially in understaffed units where workload imbalances were more prominent. Workload imbalances were attributed to unfair nursing or patient assignments, high patient-to-nurse ratios, and cancelled vacations. One nurse remarked, *“A bad day is a day where you can only provide the basics.”* Both experienced and novice nurses felt frustrated with workload imbalances. Frustration of experienced nurses was felt by being constantly assigned to complex cases, while novice nurses felt that they often handled time-consuming or challenging patients. High turnover of experienced nurses added to the nursing workload burden as skilled nurses had to train inexperienced peers, and nurses at all career stages felt pulled in different directions. Without proper training or support, inexperienced nurses lacked confidence in their roles and reported higher distress. On top of this, vacations were often denied or cancelled, and nurses felt penalized for taking sick days during the pandemic.

Allied health professionals attributed their distress to staffing shortages and increased workloads, particularly during the pandemic. They felt their supervisors did not distribute work fairly, leaving insufficient time for patient visits, chart reading, and care planning. Many perceived a lack of support from colleagues in their care team who they believed did not fully understand their roles. This group also noted that significant overtime work without flexible hours or receiving extra compensation was a source of distress. They further described challenges with inadequate coverage and access to resources when colleagues needed time off, highlighting the disparities with nursing colleagues who received support to backfill positions when there were shortages. One allied health staff stated, *“We’ve been 30% understaffed for 12 weeks. We need to address staffing disparities.”*

Physicians observed the impact of staffing shortages and workload on well-being, especially among their nursing colleagues. They recognized the connection between workload and fatigue, with one physician stating, *“Workload plays into physical and mental fatigue.”* and another stating, “*Workload without purpose leads to burnout.”* Concerns were raised about the hospital’s capacity to provide timely and accessible care, including surgeries. Physicians also felt that patient allocation was unfairly distributed, with surgeons or more senior colleagues given more opportunities to generate clinical income, leading to a sense of unfairness and workload disparities among this group.

#### Professional growth and development are key to well-being and job satisfaction

Clinicians emphasized the importance of career advancement but were uncertain how to achieve this without proper support from management and clear professional growth pathways. They also desired a more tailored approach to performance management instead of the current formulaic system. Despite valuing professional development, clinicians were concerned about the time required for such activities, given their increased clinical workloads and limited access to support, like mentorship.

Nurses stressed the importance of continuous learning for both personal and professional growth. They also emphasized the importance of team development and creating supportive environments for meaningful contributions to their profession. Nurses expressed the need for more training, participation in professional practice days, and additional support from their colleagues through mentorship. However, they found it challenging to engage in formal learning opportunities alongside their daily clinical responsibilities due to demanding workloads, staffing shortages, patient-nursing ratios, and training novice staff.

Allied health professionals were concerned about limited career advancement opportunities due to unclear professional growth pathways and limited access to job openings. They believed leadership or administrative roles were often directed toward nurses, even when allied health professionals were qualified for such positions. This group also lacked awareness of formal performance management processes to discuss their professional goals and needs, with one staff stating, “*I have not had any meetings about what I achieved or what I want to achieve.”* Without a clear path for career growth and development, some contemplated leaving their job, which created feelings of unfair treatment, favouritism, and demotivation among this group.

Physicians expressed the need for transparent, structured feedback and support by their supervisors. Many physicians acknowledged setting high standards for themselves and felt stressed by self-imposed expectations combined with institutional pressures to meet or exceed goals, which led to distress. One physician stated *“We can be happy, but not content. We can always do better and better. Even if you won the gold medal, you can get more.”* While some physicians felt supported by their teams or supervisors, they desired more opportunities for mentorship in an environment where giving or receiving support was challenging. Most physicians believed the current performance management system was ineffective. Many did not recall opportunities to openly discuss their career goals, especially at the mid-career stage, with one physician stating, *“It is hard to express my goals both personally and medically.”* Physicians also felt the need for more support in their research and educational roles. They reported being unclear about why some colleagues received more support from leadership for their professional endeavours.

#### Open and transparent leadership communication is a critical enabler of well-being

All clinician groups expressed the need for greater transparency and improvements in communication from hospital leadership. Clinicians often felt unheard at work and wanted their leaders to acknowledge the value of their input and for it to be acted upon. Some clinicians became distressed when organizational or individual level changes were not adequately communicated. All participants wanted to be more engaged throughout the change process and to be informed about the reasons behind leadership decisions.

Nurses believed that their leaders communicated important decisions ineffectively and desired more engagement and information sharing. They often lacked awareness and understanding of changes, which, coupled with expectations to comply, led to distress. Nurses recounted mixed messages across the institution about taking time off to support their well-being, especially when vacations were denied or canceled, and felt penalized for taking sick days, particularly during the pandemic. Nurses also stressed the importance of daily unit safety huddles but encountered challenges with limited inter-professional participation and discomfort expressing their ideas and concerns openly to colleagues and leaders. They found it difficult to address patient safety or workplace issues with management and often didn’t feel supported after difficult patient encounters. One nurse noted, *“There is a lack of interface with the staff as individuals.”* Some nurses hesitated to raise issues for fear of reprisal or disciplinary action. Nurses desired open and psychologically safe discussions between colleagues and management with increased presence of unit leadership on the frontlines.

Allied health professionals reported that changes were implemented in their work environment without clear communication from leadership, leading to disruptions in clinical workflows. Moreover, daily unit safety huddles and regular unit meetings were scaled back. According to one allied health professional, *“One of things I think was quite effective, when we had them, were safety huddles. Compared to staff meetings…things went up the chain of command quite quickly.”* While they appreciated safety huddles as a communication tool for addressing workplace issues, many often felt unheard by management when offering suggestions or voicing concerns, which contributed to feelings of distress. Those who felt unheard remarked on the lack of follow-up and interaction with decision-makers,which led them to believe their input was not valued, with one staff stating, *“Why can’t management deal with us…I’m not asking for mangos from an apple tree.”*

Physicians believed that hospital goals and priorities were unclear, and leadership decisions lacked transparency and effective communication about decisions related to resource allocation. This led some physicians to feel unfairly treated. Transparency was identified as a critical issue that impacted their jobs and work environments. They wanted better ways to communicate their views to leadership as they sometimes felt that their input was ignored or were uncomfortable speaking up. Physicians emphasized the need for more transparency in committee and governance structures and desired open and respectful discussions to better understand hospital decisions.

### Actionable interventions to decrease clinician distress that emerged from co-design workshops in Phase 3

Participants discussed potential intervention strategies to decrease distress and improve well-being through two guided co-design workshops. The workshops were led by project team members that are human factors specialists at our institution. Based on findings from Phases 1 and 2, participants and facilitators discussed ideas and potential strategies to mitigate identified drivers of workplace distress. Ideas and input from the workshops were then synthesized into four actionable interventions by our team.

#### Re-designing the daily safety huddle

Clinicians stressed the importance of enhancing team communication and collaboration through open and transparent discussions within psychologically safe environments. Improving daily safety huddles across clinical units emerged as a key strategy to meet this need. The redesigned huddles aim to be more impactful, engaging, and effective in addressing workplace-related factors contributing to distress while maintaining patient and healthcare worker safety as a priority. Improvements include a structured meeting template covering relevant topics, redesigned tools and materials to share information, training for effective meeting facilitation, and dedicated time and space to recognize staff. Huddles will adhere to a consistent schedule, location, and duration, promoting active engagement among inter-professional team members. This revamped approach is expected to create a safe and supportive space for teams to address relevant issues, engage in collaborative problem-solving, communicate changes, manage workloads, show respect and gratitude, and mitigate perceptions of unfair treatment in their care settings.

#### Nursing coaching and mentorship program

Clinicians highlighted a need for enhanced learning and growth opportunities within our program. Nurses reported that it was challenging to engage in career planning and learn new nursing approaches due to daily patient care responsibilities. In response to this challenge, our program will develop and launch a comprehensive coaching and mentorship program that supports the personal and professional development of cardiovascular nurses at all career stages. This program aims to improve nurses’ clinical skillset and capacity for growth by facilitating the transfer of clinical knowledge and wisdom that comes from the profession. By participating in the program, we anticipate a decrease in nursing turnover and an increase in job satisfaction, self-efficacy, and well-being with an overall improvement of retention. The program offers two educational pathways over a 16-week period: (1) clinical coaching for novice nurses (coachees) delivered by experienced nurses (coaches), and (2) mentorship for mid-career nurses (mentees) who are matched with a nursing leader (mentor). Following an 80/20 professional development model [30], nurse coach and mentee participants will be released from clinical duties to engage in professional development activities. The program will be evaluated for its feasibility and acceptability as well as the effectiveness of the intervention to improve well-being, job satisfaction, self-efficacy, and organizational commitment.

#### Value-added program e-newsletter

All clinicians expressed the importance of transparent leadership communication and the need for better awareness of organizational changes affecting their roles and work environments. The value-added program e-newsletter aims to strengthen engagement by fostering a sense of community and improving communication practices. Serving as a program-wide communication tool, the e-newsletter will provide all staff with relevant information and access to essential resources. Covering diverse topics, it will keep staff updated on program changes, with a focus on diversity, equity, and inclusion. Professional development opportunities will be shared to increase use of learning resources and enable career development. Team members will be featured to celebrate their contributions and achievements to promote a stronger sense of community and teamwork. Additional content may include information on work-life balance, employee benefits, and program-wide initiatives. To support continous organizational development, a regular feedback survey will gather input from staff for improving various aspects of their work environment. All team members will be represented and reflected in this communication and engagement strategy.

#### Employee experience e-platform

Clinicians highlighted the importance of fostering positive inter-professional relationships and creating a supportive community to improve work experiences and patient care within their teams. To achieve this, introducing a virtual employee experience platform to the unit will serve as a central hub for team engagement, collaboration, communication, and information sharing. Teams can use the e-platform to express real-time gratitude and appreciation, set individual or team goals, track progress, enhance performance, and support professional growth. The e-platform also facilitates giving and receiving constructive feedback between team members and leadership. Pulse surveys can be conducted by unit leadership to gather continuous feedback and improve the work environment, team experiences, and patient care delivery. Similar online platforms, such as BambooHR (https://www.bamboohr.com/) and Cooleaf (https://www.cooleaf.com/), are already widely used across various industries, including healthcare. It is anticipated that implementing such a platform will contribute to a more positive workplace culture by strengthening supportive inter-professional teams, promoting transparent communication, supporting professional development, and restoring joy in work.

## Discussion

The qualitative insights shared by cardiovascular clinicians highlighted various stressors in their work environment and elucidated how such factors contribute to their experiences of distress as healthcare providers. These findings not only reinforce what is known in the literature [6, 14, 18–20], but deepens our understanding about how distress manifests in different clinician groups. Qualitative discussions in Phases 1 and 2 identified five key themes: (1) supportive inter-professional teams are desired to build an effective care community; (2) joy in work is paramount for clinician well-being and exceptional patient care; (3) unsustainable workloads are strongly linked to clinician distress; (4) professional growth and development are key to well-being and job satisfaction; and (5) open and transparent leadership communication is a critical enabler of well-being. In Phase 3, the collaborative co-design process empowered clinician participants to develop tailored strategies directly addressing the workplace stressors identified. This phase proved crucial in bridging our understanding of what drives distress and collaboratively developing interventions to address these challenges with frontline clinicians. It is important to note that this project did not directly investigate strategies for unsustainable workloads or staffing shortages due to resource and time constraints. While these factors remain significant sources of clinician distress [8, 31], they are interconnected with other identified workplace stressors and should not be addressed in isolation.

Effective communication and positive inter-professional relationships among frontline clinicians and leadership emerged as critical areas for addressing interpersonal challenges, unfair workload distribution, and understanding leadership decisions impacting their work. Despite clinicians acknowledging the importance of respect and civility in the workplace, they found it challenging to uphold these values without adequate tools in a fast-paced and dynamic hospital environment. To foster a more connected workforce and enhance job satisfaction, clinicians require improved tools and resources in their work environments. To address these challenges, our team plans to re-design existing communication mechanisms, including safety huddles and the program e-newsletter, while introducing new platforms such as the employee experience e-platform. The safety huddle is known as a powerful tool for enriching communication, collaboration, and coordination among frontline workers while also improving job satisfaction [32, 33]. Existing safety huddles will be redesigned to create interdisciplinary and psychologically safe spaces for discussing patient and worker safety issues within teams [34].

Moreover, we will implement a value-added e-newsletter and employee experience e-platform as complimentary strategies to strengthen communication and engagement of healthcare workers and leadership across the program. Engaging staff is essential for a high-performing healthcare organization. Strengthening engagement with leadership, establishing two-way communication channels, and bringing staff recognition to the forefront are a means to improve inter-professional relationships, social connectedness, and job satisfaction among healthcare workers [35, 36]. Connectedness and positive support are also critical enablers for improved physical and mental health of healthcare workers [36], requiring more attention to strengthen relationships among teams and foster a sense of belonging. These strategies aim to promote clinician well-being by cultivating a workplace culture that encourages open and transparent communication within a diverse community of healthcare workers through planned and structured communications. At the same time, mutual trust and respect among healthcare workers must be nurtured to improve inter-professional relationships and collaborative teamwork. Leveraging these tools can help build a culture within healthcare teams that fosters social connections while promoting trust, respect, and belonging. Reinforcing these values is integral to the success of these interventions, forming the foundation for quality communication, effective inter-professional care teams, and positive work environments.

In addition, sharing a desire for a workplace that is more connected and engaged, all clinicians emphasized the importance of continuous learning and professional growth. Learning and development play a critical role in promoting job satisfaction and retention among healthcare workers [14, 37, 38]. However, clinicians in this project highlighted the challenges in accessing professional development opportunities due to heavy workloads and time constraints during daily patient care activities. Limited access or support for professional development can create a lost sense of meaning and purpose in work and leave clinicians feeling undervalued for their efforts [39–41]. These challenges were particularly concerning for nurses who were dramatically affected by staffing challenges and increased clinical demands, especially during the COVID-19 pandemic. Job satisfaction is a significant predictor of burnout and distress among nurses [19, 42], and as the troubling number of nurses leaving hospital settings continues to rise, investing in the nursing workforce remains critical [43, 44]. To address this, introducing formal coaching and mentorship was proposed as a strategy to enable professional growth and improve job satisfaction of nurses. Mentorship is a well-documented strategy to improve retention and job satisfaction within this profession [38]. Implementing a formal nurse coaching and mentorship program is expected to reduce turnover and positively impact nursing work experiences and delivery of patient care. Understanding the clinical demands and time constraints faced by nurses, we determined that nurses needed protected time to engage in professional development activities, which requires release time from their clinical duties. Through the implementation of a formalized program, we have proposed using an 80 − 20 professional development model that enables nurses to devote one shift per week to professional development activities. This model has proven successful in reducing sick and overtime hours, increasing provider and patient satisfaction, and sustaining time for education [30]. The program aims to bridge the gap between nurses’ desire for ongoing learning and their limited capacity for professional development. By focusing on opportunities for professional growth and redesigning work experiences of nurses, we aim to shift the collective mindset from crisis care to a sustainable model that fosters continuous learning and growth within the hospital environment.

The shared experiences and unique challenges uncovered by cardiovascular nurses, allied healthcare professionals, and physicians highlights the importance of exploring workplace-related factors that contribute to clinician distress from different provider perspectives. This initiative also reinforces the critical need for healthcare organizations to continuously assess healthcare worker well-being and to actively engage inter-professional clinicians as true partners in the identification and development of targeted interventions to mitigate distress in the workplace.

### Limitations

Our results should be interpreted within the limitations of the study design. Findings and interpretations were based on a subset of clinicians within a diverse workforce in a cardiovascular care program at a large quaternary healthcare centre and may not be generalizable. Interviews focused on the experiences of clinician participants working in cardiovascular care specialities and may not account for contextual sensitivities in other care settings. This project took place during the COVID-19 pandemic, potentially affecting participation rates and sources of distress identified. It is worth noting that unsustainable workloads and staffing shortages were not directly addressed in the co-design workshops as these workplace-related factors require further investigation, action, and investment at the organization and health system level.

### Implications

Developing intervention strategies to mitigate clinician distress may require organizations to make drastic culture shifts to cultivate healthier workplaces and promote well-being. Critical steps organizations can take to address clinician distress is to first asses healthcare worker well-being. Second, directly engage clinicians to understand how workplace-related factors contribute to distress and burnout. Third, collaboratively design interventions or strategies with those impacted to address workplace sources of distress. Using findings from this work, organizations may choose to focus on fostering positive inter-professional relationships, reinforcing effective communication, building capacity for professional development, and recognizing and rewarding staff. This approach aligns with recommendations from the National Academy of Medicine [1], and provides a path for hospital leadership to proactively improve the well-being of their workforce.

## Conclusion

Findings from this work underscore that hospital work environments are major sources of distress for clinicians, as described by participating cardiovascular nurses, allied health professionals, and physicians. Healthcare organizations must develop effective intervention strategies aimed at mitigating clinician distress and improving well-being. This can be achieved by actively engaging healthcare workers as genuine partners to comprehensively assess well-being and collaboratively design tailored and practical interventions that directly address sources of workplace distress. 

### Electronic supplementary material

Below is the link to the electronic supplementary material.

Supplementary Material 1

## Data Availability

Data and materials of this work are available from the corresponding author upon reasonable request: ahlexxi.jelen@uhn.ca.

## Abbreviations

PMCC: Peter Munk Cardiac Centre
