# Supplementary material for: A qualitative co-design-based approach to identify sources of workplace-related distress and develop well-being strategies for cardiovascular nurses, allied health professionals, and physicians
Source: BMC Health Serv Res. 2024 Feb 26;24:246. doi: 10.1186/s12913-024-10669-x (PMC10897985; doi:10.1186/s12913-024-10669-x)
Supplement: Supplementary file 1 — Supplementary Material 1 [file 12913_2024_10669_MOESM1_ESM.docx]

**Supplement.** Discussion Guides for Phase 1-3

**Phase 1 Discussion Guide: One-on-one interviews (45 minutes)**

**Purpose/Consent Introduction:**

Many health care workers suffer from burnout, a work-related syndrome characterized by emotional exhaustion, a sense of reduced personal accomplishment, and depersonalization. Health care workers may also experience distress, a composite that includes decreased meaning in work, severe fatigue, unbalanced work–life integration, low quality of life and suicidal ideation.

At the Peter Munk Cardiac Centre (PMCC), our focus is to better understand how the perception of unfair treatment drives burnout and distress among PMCC clinicians, and on developing intervention strategies that will decrease the perception of unfair treatment in the workplace. This approach is essential because decreased burnout and distress among our team will improve each of our individual work experiences and well-being. With COVID-19 impacting us all, the well-being of our employees is ever more important.

The results of our previous quality initiative, supported by the PMCC Innovation Fund, show that burnout is a significant challenge that must be addressed. Specifically, this research showed that 78% of nurses, 65% of physicians and 56% of allied health staff had high levels of distress. Among all three groups, the perception of unfair treatment at work emerged as the principal driver of PMCC clinician burnout and distress.

Our quality improvement study on well-being will take place over several weeks and will involve virtual one-on-one interviews, focus groups and co-design intervention workshops. This approach allows us to build on the learnings from our quantitative Well-Being Index Survey Assessment. This initiative is part of a continuous process to learn about individual work experiences and to build on those learnings to design real world interventions that support all of our staff to improve their well-being at work.

Feedback provided in interviews and focus groups will be kept confidential and will not identify you or impact your employment at UHN. All interview and focus group responses will be reported at an aggregate level, and you will not be named in any reports or publications that result from this initiative. Only high-level themes arising from interviews and focus groups will be summarized and shared with clinical leadership. The purpose of these interviews is to increase our understanding of what is meant by unfair treatment at work, and to develop and validate intervention strategies that will reduce unfair treatment and decrease the prevalence of clinician burnout and distress in the PMCC. Findings may also be shared through publications or conferences so that other centres can implement strategies to mitigate burnout and support the well-being of their staff.

Confidential interview data will be stored under a double locked system with the PMCC project team, Barry Rubin and Leanna Graham, for up to 5 years. This information will be kept separately from any employment records and only members of the PMCC project team will have access to it now and in the future.

This interview is **optional.** If you decide not to participate, your employment will not be affected. By participating in this interview, you are giving consent to use the information provided as a way to better understand how the perception of unfair treatment drives burnout and distress among PMCC clinicians, and on developing intervention strategies that will decrease the perception of unfair treatment in the workplace.

If you have questions about this QI project, please contact [ahlexxi.jelen@uhn.ca. If you have questions about your rights as a participant in a UHN Quality Improvement Project, please contact the UHN Quality Improvement Review Committee (QIRC) at QI@uhn.ca. QIRC is a group of people who oversee the ethical conduct of QI projects; they are not part of the project team.

**Introduction (5 min)**

- - - - Explain process (confidentiality, purpose of research).
      - Introduce self – include a little about profession, family, current living environment/work life re COVID
      - Please tell me a little about what yourself (PROBE: How have you/and your family been coping with COVID-19? What are your biggest challenges/hopes?).

**PMCC – General (15 min)**

- - - - We will be spending the next while talking about you and your role as a [insert job/role] at PMCC.
      - How long have you been working at PMCC?
      - What do you do in your role? (PROBE: What do you most enjoy about it? What do you like least?)
      - What is typical day like for you? What are the best parts? Worst parts?
      - And what about now during COVID-19, how does COVID impact your work generally? Each day?
      - Please tell me how you might summarize your experience at PMCC.
      - What about burnout and distress? We know from our research that burnout and distress are real issues that we need to address at PMCC.
      - What are your thoughts on burnout? On distress? PROVIDE DEFINITIONS AS NEEDED
    - Burnout is a work-related syndrome characterized by emotional exhaustion and a sense of reduced personal accomplishment.
    - Distress is a composite of burnout, meaning in work, severe fatigue, work–life integration, quality of life and suicidal ideation
  - How do you think they affect the team at PMCC? (PROBE: doctors, nurses and allied health as relevant.)
  - I know that this can be difficult to talk about, but how do burnout and distress affect you specifically?
  - And how does COVID affect all of this?
  - And what discrimination? (PROBE: Have you experienced racial discrimination? Gender discrimination? Do you think this contributes to burnout and distress?)

**PMCC – Specific (20 min)**

We believe that addressing the issue of fairness is important and can have an impact on decreasing burnout and distress. I would like to focus on some specific questions about fairness.

- Thinking as generally as possible, what is fairness to you? What does it mean to be treated fairly? Unfairly?
- For you, what is the link between fairness and well-being? Are they connected? How?
- What is fairness at PMCC?
- In what situations are individuals working at PMCC treated fairly? Unfairly? (PROBE: doctors, nurses and allied health as relevant.)
- Again, I know it can be hard to personalize, but what is your own perception of how you are treated? What situations do you feel you are treated fairly? Unfairly? And how does this connect to your own well-being?

PROBE on all hypothesis:

- What about workload? (PROBE: division of tasks, breaks, assignment of difficult patients, competing demands of patients/families, not being assigned a lead role, shifts, other demands of time, anything else?)
- Being heard? (PROBE: ability to communicate individual wants and needs, ability to communicate issues about effectively delivering the best care, communications of safety/problems, not being heard, feeling disrespected or listened to, anything else?)
- Management of change? (PROBE: Communications on new policies/procedures, training, knowledge for successful implementation, anything else?)
  - PROBE: EPIC
- Performance Management: (PROBE: understanding of current system and how it works, perception of fairness, ability to express career goals, ability to advance career, anything else?)
- Other as relevant
- Do you think your position or role relates to fairness? Would it matter if you had a different role?
- We, as you know, are looking for real solutions or interventions. What do you think can be done to address [insert as appropriate based on discussion]? (PROBE: specifics as relevant)

**Conclusion (5 min)**

- We are about complete, is there anything else that we have not discussed that you think is important?

Thank you for your time and for sharing information.

**Phase 2 Discussion Guide: Focus Groups (90 minutes)**

**Purpose/Consent Introduction:**

Many health care workers suffer from burnout, a work-related syndrome characterized by emotional exhaustion, a sense of reduced personal accomplishment, and depersonalization. Health care workers may also experience distress, a composite that includes decreased meaning in work, severe fatigue, unbalanced work–life integration, low quality of life and suicidal ideation.

At the Peter Munk Cardiac Centre (PMCC), our focus is to better understand how the perception of unfair treatment drives burnout and distress among PMCC clinicians, and on developing intervention strategies that will decrease the perception of unfair treatment in the workplace. This approach is essential because decreased burnout and distress among our team will improve each of our individual work experiences and well-being. With COVID-19 impacting us all, the well-being of our employees is ever more important.

The results of our previous quality initiative, supported by the PMCC Innovation Fund, show that burnout is a significant challenge that must be addressed. Specifically, this research showed that 78% of nurses, 65% of physicians and 56% of allied health staff had high levels of distress. Among all three groups, the perception of unfair treatment at work emerged as the principal driver of PMCC clinician burnout and distress.

Our quality improvement study on well-being will take place over several weeks and will involve virtual one-on-one interviews, focus groups and co-design intervention workshops. We have completed the one-on-one interviews, and are looking for participation in the focus groups. Our approach allows us to build on the learnings from our quantitative Well-Being Index Survey Assessment. This initiative is part of a continuous process to learn about individual work experiences and to build on those learnings to design real world interventions that support all of our staff to improve their well-being at work.

Feedback provided in interviews and focus groups will be kept confidential and will not identify you or impact your employment at UHN. All interview and focus group responses will be reported at an aggregate level, and you will not be named in any reports or publications that result from this initiative. Only high-level themes arising from interviews and focus groups will be summarized and shared with clinical leadership. The purpose of these interviews and focus groups is to increase our understanding of what is meant by unfair treatment at work, and to develop and validate intervention strategies that will reduce unfair treatment and decrease the prevalence of clinician burnout and distress in the PMCC. Findings may also be shared through publications or conferences so that other centres can implement strategies to mitigate burnout and support the well-being of their staff.

Confidential interview data will be stored under a double locked system with the PMCC project team, Barry Rubin and Leanna Graham, for up to 5 years. This information will be kept separately from any employment records and only members of the PMCC project team will have access to it now and in the future.

Participation in this focus group is **optional.** If you decide not to participate, your employment will not be affected. By participating in this focus group, you are giving consent to use the information provided as a way to better understand how the perception of unfair treatment drives burnout and distress among PMCC clinicians, and on developing intervention strategies that will decrease the perception of unfair treatment in the workplace.

If you have questions about this QI project, please contact ahlexxi.jelen@uhn.ca. If you have questions about your rights as a participant in a UHN Quality Improvement Project, please contact the UHN Quality Improvement Review Committee (QIRC) at QI@uhn.ca. QIRC is a group of people who oversee the ethical conduct of QI projects; they are not part of the project team.

**Introductions + Icebreakers (10 minutes)**

- *Explain process (confidentiality, purpose of research)*
- *Introduce self – include a little about profession*
- Please tell me a little about what yourself (PROBE: as appropriate remembering that individuals may be less personal in a group setting: name, role, years worked at PMCC)
- *Icebreaker activity (as appropriate depending on participants)*

**Overview: Interview Findings and Broad Discussion (35 minutes)**

- *Presentation of key findings from interviews*

1. **Key Drivers of Joy**

- All are proud to work at an institution that is considered the fourth best in the world. Clinicians see the Peter Munk Cardiac Centre as a great place to achieve their professional goals. All take pride in making a difference in the lives of people they care for.

1. **Performance Management**

- Most believe that there is no clear path for growth or achieving professional goals. Staff report limited awareness for performance management. Most do not recall meetings formal or informal about personal professional performance and growth.

1. **Workload**

- Clinicians report that division of work is not always fairly divided and at times feel that colleagues are given preferential treatment. All report that workload has increased because of COVID-19.

1. **Management Support**

- Clinicians express a challenge ‘feeling heard’. Change at the organizational and individual level is a cause of distress. There are general feelings that reasons for change are not always well communicated and that it is difficult to give input on both potential and existing changes.
- Many feel that both formal and informal communications are not always delivered respectfully.
- Team meetings are cancelled or missed because of COVID-19.
- Gratitude is not always purposefully articulated. Clinicians say even a simple ‘thank you’ makes them feel valued and appreciated. They want to really feel appreciated through willingness to solve issues.

1. **Inter-professional Relationships**

- All see inter-professional relationships as an opportunity for improvement. All have empathy for nurses and the difficult staffing situation that they are experiencing. Some clinicians identify disrespectful interactions with colleagues as a real concern.

1. **Discrimination**

Most do not report racial or gender discrimination as a challenge at PMCC or UHN. Specifically, some see favouritism or barriers related to age, experience level, health, culture and language.

We will be spending the next while talking about burnout and distress at PMCC. We know from our survey research and one-on-one interviews that burnout and distress are real issues that we need to address at PMCC. Our survey revealed that being treated unfairly was a driver of burnout. Our interviews helped us get a better understanding of how fairness unfair treatment drives burnout. During our discussion, we would like to explore some of our learnings, what they mean to you, and how you think these issues could be mitigated through possible interventions.

Before we begin, I would like to review the definitions of burnout and distress:

- Burnout is a work-related syndrome characterized by emotional exhaustion and a sense of reduced personal accomplishment, and depersonalization that may manifest as negativity, cynicism and the inability to express empathy or grief.
- Distress is a composite of burnout, meaning in work, severe fatigue, work–life integration, quality of life and suicidal ideation.
- Six key themes emerged from the interview. I would like to review generally what we heard.
- *Open it up to the group with the following questions:*
- Do any of these findings surprise you?
  - - Probe by theme: Joy, Performance Management, Workload, Inter-professional relations, and discrimination
- Do they resonate with you? Why/why not?
  - - Probe by theme: Joy, Performance Management, Workload, Inter-professional relations, and discrimination
- Is there something you feel is important that wasn’t touched on?

**Ideation + Mitigation Design (40 minutes)**

- *Presentation of brainstorm questions (that stem from key one-on-one interview findings) and will create the foundation for the remainder of the discussion. Questions may be added or deleted depending on group input.*
- Two-way communication*: How might we improve communication across the unit?*
  - - To ensure that staff feel heard.
    - To ensure that leadership decisions are understood.
    - Probe: person-to-person, email, meetings, seeing manger
- Respect as a way of doing things: *How might we create a culture of respect?*
  - - How does gratitude fit in?
    - Recognition?
    - Empathy and kindness?
    - Does favouritism place a role?
    - Task allocation
    - How might we create a just culture?
    - Probe: Workload and pressure leaving people like they do not have the resources for the extra effort to deliver respect/gratitude
- Professional growth: *How might we better support professional and personal development?*
  - - Reasons for joy at work
    - Regular team meetings
    - Meeting for professional and personal development/performance reviews
    - Getting growth-oriented feedback
    - Speaking-up
- *Facilitator notes:* ***Spend approximately 10-15 minutes per brainstorm question****. Review brainstorm question and prompts*
  - - *Rapidfire ideation: participants are given a few minutes to jot down the ideas that immediately come to mind and then share with the group.*
    - *Probe round: participants are provided with probes (concepts / ideas) and given the chance to respond to them*

**Conclusion (5 min)**

We are about complete, is there anything else that we have not discussed that you think is important?

Thank you for your time and for sharing information.

**Phase 3 Facilitator’s Guide: Co-design workshop (120 minutes)**

**Opening Remarks (5 minutes)**

- Welcome everyone + introduce HHF as facilitators
- Leadership message
- Overview of the session
- Objectives
- Agenda
- Ground rules for the day

**Understanding Burnout at PMCC (15 minutes)**

- Overview of the research done today
- Methods: survey + interviews + focus groups
- Key Findings
- Focus for Today’s Workshop
- Discussion

**Move to breakout rooms, introductions, and icebreaker (5 minutes)**

Facilitators to ask each participant to introduce themselves + their role + one icebreaker question from the list below

- If you were a dessert what would you be and why?
- If you could be any animal, what would you be and why?
- If you were stranded on a desert island and had the option of bringing three items with you, what three items would they be?
- What was the first concert you ever went to?
- If you could have any celebrity over for dinner, who would it be and why?

**Breakout Brainstorm (90 minutes)**

***Ideate! The objective here is to generate as many ideas as possible***

- *Let participants know that this is no judgement zone - there will be time later for us to prioritize ideas by their value/feasibility*
- *15 minutes per brainstorm question*
  - *1 minute - Facilitator to review the question*
  - *3 minutes - Give participants time to individually jot down ideas*
  - *5 minutes - Share ideas → Facilitator to capture on Miro board stickies*
  - *3 minutes - Ask participants to use their digital deck of cards to look for inspiration and generate at least one more idea*
  - *3 minutes - Share ideas → Facilitator to capture on Miro board stickies*
- ***Affinity Map + Prioritize***
  - *7 minutes per brainstorm question*
  - *2 minutes - Facilitator to thematically group ideas (if not done already)*
  - *5 minutes - Move clusters/ideas to PICK chart*

*Facilitation note: if all the ideas in the cluster are similar, move the cluster as a whole. However, if some ideas are distinct, map them to the PICK chart individually.*

***Ask Group Which Two Ideas They’d Like to Share Back***

***Return to Main Room***

**Shareback and Close (5 minutes)**

- Each group to share their top two ideas
- Comment on next steps
- Workshop adjourns
